# Chronic recalcitrant erythema nodosum leprosum: therapeutic dilemma and role of *mycobacterium indicus pranii* vaccine^☆^

**DOI:** 10.1016/j.abd.2020.08.032

**Published:** 2021-11-27

**Authors:** Sunil Kumar Gupta, Sushantika Kumari

**Affiliations:** Department of Dermatology, All India Institute of Medical Sciences, Gorakhpur, India

**Keywords:** Erythema nodosum, Leprosy, Mycobacterium infections, Vaccines

## Abstract

Erythema nodosum leprosum is a severe immune reaction that complicates the usual course of multibacillary leprosy. There is increased activation of T-cells in erythema nodosum leprosum. Treatment modalities available to date for the management are systemic steroids, thalidomide, methotrexate, cyclophosphamide, azathioprine, minocycline, and apremilast but none of them is promising and safe. *Mycobacterium indicus pranii* is an atypical mycobacterium possessing strong immunomodulatory properties. The vaccine for this mycobacterium has been shown to have both immunotherapeutic and immunoprophylactic effects in multibacillary leprosy patients. We report a case of chronic recalcitrant erythema nodosum Leprosum which responded to *Mycobacterium indicus pranii* vaccine without any adverse effects, thereby suggesting its role as a novel therapeutic option in this reaction.

## Introduction

Leprosy is an infectious disease caused by *Mycobacterium leprae*. The disease affects the peripheral nerves, the skin, the mucosa of the upper respiratory tract, and the eyes. The indolent course of the disease is interrupted by acute outbursts termed as Lepra reactions (reversal reactions and Erythema Nodosum Leprosum [ENL]). ENL is characterized by crops of tender papules and nodules with high-grade fever and other constitutional symptoms. The management of chronic recalcitrant ENL is difficult and often requires systemic agents like corticosteroids, thalidomide, clofazimine, minocycline, or immunomodulators either alone or in combination for many months.^1^ *Mycobacterium Indicus Pranii* (MIP) vaccine has been shown to have both immunotherapeutic and immunoprophylactic effects in multibacillary leprosy patients.^2^ It also reduced the bacillary load, upgraded the lesions immunohistologically, led to complete clearance of granuloma, and reduced the duration of Multidrug Therapy (MDT) in leprosy patients.

This vaccine has not been tried so much in patients with reactions in leprosy as there was a risk of precipitating severe reactions post-vaccination. In this case, the authors of the present study tried the vaccine in a patient of ENL who was steroid-dependent and refractory to thalidomide and other second-line drugs and found a very satisfactory result with a single dose of the MIP vaccine.

## Case

**Figure 1:**
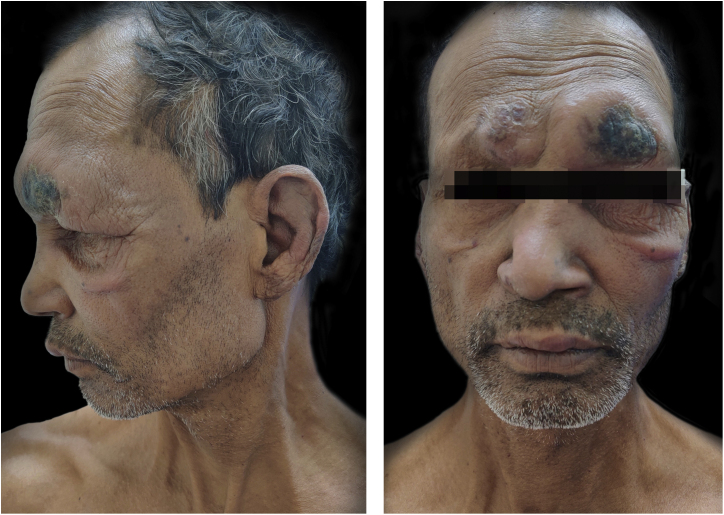
Lepromatous Leprosy with ENL showing lesions before vaccination.

A 55-years-male came to Dermatology outpatient with a history of Hansen’s disease for which the patient had been taking MDT for one and a half years. For the last 8 months, the patient had been developing fever with tender evanescent nodules over the body. General body examination also revealed diffuse infiltration with supraciliary madarosis (Fig. 1). Sensory examination showed a glove and stocking pattern of hypoesthesia. Neurological examination showed bilateral symmetrical mild thickening of ulnar and common peroneal nerves but no tenderness and power in hands and feet muscles were within normal limit. The patient was diagnosed as a case of Lepromatous Leprosy with ENL. Routine investigations were within normal limits except leukocytosis, and Bacteriological Index (BI) 5+. According to The Erythema Nodosum Leprosum International Study (ENLIST) severity scale, (pain = 3, fever = 3, lesions = 2, inflammation = 3, extension of lesion = 2, peripheral edema = 1, bone pain = 1, arthritis = 1, lymphadenopathy = 0 and neuritis = 0) score was 16.^3^

**Figure 2:**
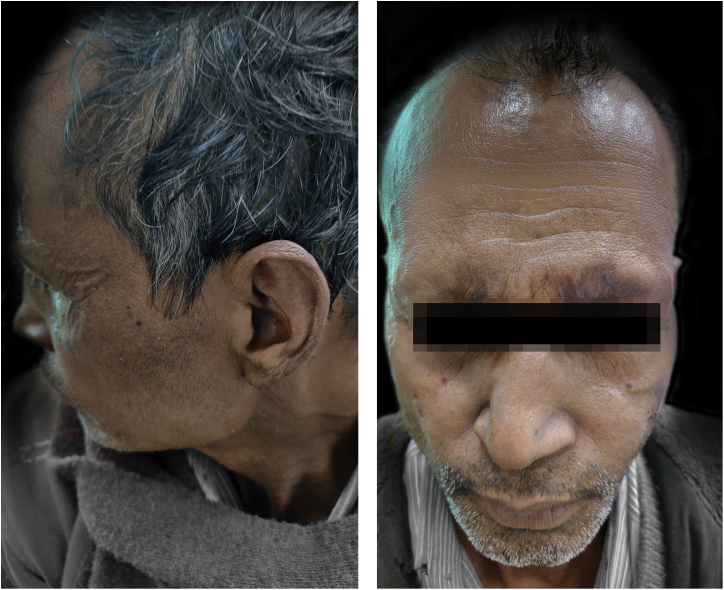
Lepromatous Leprosy with ENL showing clearance of lesions after MIP vaccination.

The patient was started on taking Thalidomide 300 mg/day, MB-MDT with 300 mg/day of clofazimine, methylprednisolone 32 mg, and analgesics to relieve pain. After two weeks, the patient started developing ulnar neuritis with increase numbness in both hands and feet. So, Thalidomide was stopped, and minocycline 100 mg was started. But there was no improvement in ENL after a month. Then the patient was also put on oral methotrexate 15 mg/week. Even after four weeks of treatment with a combination of methylprednisolone, methotrexate, minocycline, analgesic, and MB-MDT, the patient’s condition remained the same with frequent crops of lesions. Then the authors stop all the drugs except MDT and analgesics and planned MIP vaccination after taking consent. The patient was given MIP vaccine, 0.1 mL intradermally in both arms (around deltoid muscle insertion). The patient did not complain of any side effects due to vaccination. The condition was much improved after two weeks (Fig. 2). Afterward, the patient had put on minocycline boosted MDT and analgesics for the next 6 months. On further follow-up (after total treatment duration of 2 years and 3 months), the patient was evaluated clinically, and BI was decreased to 4+ with no recurrence.

## Discussion

The WHO launched a 5-year “Global leprosy strategy 2016–2020” in April 2016 titled “accelerating towards a leprosy-free world”. Based on total cases at the end of 2018, the prevalence rate of Leprosy corresponds to 0.2/10,000 according to WHO.^4^ The incidence of ENL in patients with multibacillary leprosy is up to 24%. ENL can occur before, during, or after antileprosy treatment, but it is most common in the first 6 months of treatment.

ENL is an immune-mediated inflammatory complication. ENL occurs due to the release of proinflammatory mediators such as Tumor Necrosis Factor (TNF)-alpha, Interferon (IFN)-gamma, and Interleukin (IL)-2, IL-6 and IL-12, IL-17.^5^ There is increased activation of T-cells in entreated ENL. The ratio of regulatory T-cells to effector memory T-cell decreased in ENL in comparison to LL controls and more T cells are antigen-experienced in ENL.^6^

High-dose corticosteroid is the mainstay of the treatment of ENL but prolonged use leads to serious adverse effects and steroid dependence. The second-line drugs like thalidomide, clofazimine, pentoxifylline, cyclophosphamide, and methotrexate are also very effective and used as steroid-sparing agents.^1^ Sometimes chronic ENL becomes refractory to second-line drugs and poses a therapeutic dilemma. A literature search on PubMed revealed the role of azathioprine, Tumor Necrosis Factor (TNF)-α inhibitors, minocycline, and apremilast, in the management of chronic recalcitrant ENL but some of these are associated with unacceptable adverse effects, longer duration and high cost of treatment (Table 1).^7^, ^8^, ^9^

**Table 1:** Enlisting the drugs tried in chronic recalcitrant ENL.

Drug	Reference	Study type	Dose & duration	Patient’s number	Type of leprosy & duration	ENL refractory to drugs	BI-Pre/Post treatment
Infliximab	Faber WR et al. N Engl J Med. 2006;355:739-739	Case Report (Corresponds)	300 mg i.v. on week 1, 2, & 6.	52-year female	BLHD-18 months	Prednisolone	5+/not available
						Thalidomide	
						Pentoxyfillin	
Etanercept	Michele L et al. Clinical Infectious Diseases, 2011;52(5):e133-135	Case Report	50 mg/week s.c. × 2 years	33-year female	LLHD-2 months	Prednisone	2+ to 4+/ not available
						Thalidomide	
						Clofazimine	
	Chowdhry S et al. Int J Mycobacteriol,2016;5(2):223-225	Case Report	50 mg/week s.c. × 16 weeks	49-years male	LLHD-2 months	Prednisolone	6+/Not available
						Clofazimine	
						Thalidomide	
						Minocycline	
						Clarithromycin	
						Oflaxacin	
						Pentoxyfilline	
						Azathioprine	
	Santos JRS et al. An Bras Dermatol. 2017;92(4):575-577	Case Report	50 mg/week s.c. × 11 months	40-years male	LLHD-1 year	Prednisolone	Not available
						Thalidomide	
Minocycline	Narang T et al JAMA Dermatol. 2015;151(9):1026-1028.	Prospective Pilot Study	100 mg/day × 3 months	10 patients	8 LLHD+ 1 BLHD+ 1 Histoid for last one year	Prednisolone	2+/ decrease by 1 log
						Clofazimine	
						Thalidomide	
						Pentoxyfillin	
						Colchicine	
						Hydroxychloroquine	
						Azathioprine	
Azathioprine	Jitendra SSV et al. J Clin Diagn Res. 2017;11(8)	Case Reports	100 mg/day × 12 months	48-years male	LLHD-4 years	Prednisolone	6+/Not available
						Clofazimine	
						Thalidomide	
Apremilast	Narang T et al. Br J Dermatol. 2020;182(4):1034-1037.	Case Report	30 mg BID following standard dose escalation in first week × 5 months	2	LLHD-8 months	Prednisolone	Not available
				34-years male		Clofazimine	
						Colchicine	
				31-years male	LLHD-12 months	Minocyclin	
						Pentoxyfillin	
						Thalidomide	

LLHD, Lepromatous Hansen Disease; BLHD, Borderline Lepromatous Hansen Disease; ENL, Erythema Nodosum Leprosum; BI, Bacteriological Index; i.v., Intravenous; s.c., Subcutaneous.

MIP is an autoclaved suspension of non-pathogenic mycobacteria. It modulates the cellular immune response towards the protective Th1 type. It converts nearly 98% of normal lepromin negative healthy contacts to lepromin positivity status.^2^ Earlier, the vaccine was avoided in reactions of Leprosy as it was suspected to precipitate one itself. But the authors’ observation on using a single dose of the MIP vaccine in chronic recalcitrant ENL gives an insight in clearing out the acute reaction state of the disease as well. ENL is triggered when there is an abundance of fragmented or granular bacilli in tissues. It has been also postulated that in ENL, there is an imbalance of immunoregulatory T-cell subsets. This is manifested as an increased ratio of helper (CD4+) to suppressor/cytotoxic (CD8+) T-cells in the blood. A decrease in CD8+T-cells may favor the formation and deposition of immune complexes. After immunization with the MIP vaccine, there is accelerated bacteriological clearance and induction of IFN-γ, TNF-α, and IL-12 secretion, higher NK cell and CD8+ T-cell cytotoxic activity, and a decrease in B cells recruitment.^10^ This is a possible explanation of the role of MIP in ENL.

As in ENL, the authors have some limited therapeutic options, adding this vaccine to some treatment-resistant cases will only help us in the future. Though this case suggests that MIP is not just a preventive modality, also as add-on therapy with other anti-ENL regimens but further randomized controlled trials will be needed to explore more possibilities with this vaccine.

## Financial support

None declared.

## Authors’ contributions

Sunil Kumar Gupta: Conception and design of the study, acquisition of data, analysis and interpretation of data; drafting the article.

Sushantika Kumari: Conception and design of the study, acquisition of data, analysis and interpretation of data; drafting the article.

## Conflicts of interest

None declared.
